# “Vision Loss” and COVID-19 Infection: A Systematic Review and Meta-Analysis

**DOI:** 10.3390/vision6040060

**Published:** 2022-09-23

**Authors:** Matteo Ripa, Lorenzo Motta, Chiara Schipa, Stanislao Rizzo, Liliana Sollazzi, Paola Aceto

**Affiliations:** 1 Ophthalmology Unit, Fondazione Policlinico Universitario A. Gemelli IRCCS, 00168 Rome, Italy; 2 Catholic University “Sacro Cuore”, 00168 Rome, Italy; 3 Department of Ophthalmology, William Harvey Hospital, East Kent Hospitals University NHS Foundation Trust, Ashford TN24 0LZ, UK; 4 Department of Emergency, Anesthesiological and Reanimation Sciences, Fondazione Policlinico Universitario A. Gemelli IRCCS, 00168 Rome, Italy; 5 Consiglio Nazionale delle Ricerche, Istituto di Neuroscienze, 56127 Pisa, Italy

**Keywords:** COVID-19, poor vision, visual impairment

## Abstract

Background: Visual impairment in terms of reduced visual acuity and “visual loss” has been reported as an atypical symptom in patients with severe acute respiratory syndrome coronavirus-2 (SARS-CoV-2) infection. This systematic review and meta-analysis aims to assess the cumulative incidence of “visual loss” during coronavirus disease 2019 (COVID-19) and review the current evidence regarding “visual loss” caused by SARS-CoV-2 infection. Methods: We performed a systematic review and meta-analysis of studies following Preferred Reporting Items for Systematic Reviews and Meta-Analyses guidelines. We systematically searched the PubMed, Embase, and Scopus databases for relevant studies published that clearly described “vision loss” and SARS-CoV-2 infection. All studies reporting concomitant “vision loss” and laboratory-confirmed SARS-CoV-2 infection were included. Meta-analyses were conducted using the measurement of risk and a 95% confidence interval for each study. Results: Our search identified 1143 manuscripts published in the English language. After study screening, twenty-nine articles were selected: two cross-sectional studies, twenty-four case reports, and three case series. A random-effect meta-analysis demonstrated that the pooled “visual loss” cumulative incidence in COVID-19 patients was 0.16 (95% CI 0.12–0.21). The quality rating of the cross-sectional studies averaged four out of the maximum score on the Newcastle–Ottawa scale. Conclusions: COVID-19 infection might cause “visual loss”. Even if the current evidence is limited, ophthalmological assessment should be promptly provided to all patients experiencing visual impairment symptoms during SARS-CoV-2 infection.

## 1. Introduction

Severe acute respiratory syndrome coronavirus-2 (SARS-CoV-2) is a single-stranded RNA virus that belongs to the Coronaviridae family [1].

The World Health Organization (WHO) declared the coronavirus disease 2019 (COVID-19) outbreak a global pandemic on 11 March 2020, which led to a significant economic and healthcare burden [2]. Current available diagnostic tests to detect COVID-19 include a triad of complementary approaches. The polymerase chain reaction (PCR) is the most highly sensitive and specific molecular test to detect SARS-CoV-2 nucleic acids’ presence, representing the gold standard technique because of its sensitivity and specificity. It uses primers matching a segment of the SARS-CoV-2 genetic material to detect COVID-19 [3].

After exposure, the average incubation period may range from four to five days [4]. A wide range of symptoms has been associated with the SARS-CoV-2 infection, whose severity may vary from asymptomatic to death. Although most patients either remain asymptomatic or experience common viral infection symptoms such as fever, cough, and fatigue, some patients may develop atypical symptoms such as neurological (headaches, loss of taste, smell) and ophthalmological symptoms (conjunctivitis, epiphora, and “vision loss”) [5]. Based on multiple cross-sectional studies, the incidence of ocular manifestation in COVID-19 patients might be as high as 30% [6]. At the beginning of the pandemic, many physicians reported eye redness and irritation in patients, describing “conjunctival congestion” in Wuhan, China. In a recent systematic review and meta-analysis, Inomata et al. reported clinical and prodromal ocular symptoms in patients with COVID-19. The most common ocular findings among COVID-19 patients were conjunctivitis (86.4%), ocular pain (34.4%), dry eye (33.3%), and floaters (6.7%) [7]. ”Visual loss” in COVID-19 patients was reported in a few articles. Nonetheless, it has been observed that its onset may be due to viral neurotropism and indirect immunologic and neurovascular effects [8,9,10,11,12,13,14,15,16,17,18,19,20,21,22,23,24,25,26,27,28,29,30,31,32,33,34,35,36].

Furthermore, despite extensive research on sensory manifestations of COVID-19 since the start of the pandemic, only a few articles and no meta-analysis papers have assessed “vision loss” as a symptom during the SARS-CoV-2 infection.

The present paper intends to systematically review the current evidence regarding “visual loss” caused by SARS-CoV-2 and to determine its cumulative incidence through a meta-analysis. In addition, we further aimed to identify the characteristics of the “visual loss”, thus evaluating factors that could contribute to understanding the association between COVID-19 and “visual loss”.

## 2. Materials and Methods

### 2.1. Search Strategy

Three databases (PubMed, Embase, and Scopus) were checked from inception until 9 June 2022, using free text and controlled vocabulary (MeSH or Emtree) to analyze the relationship between visual impairment and SARS-CoV-2 infection.

**Figure 1:**
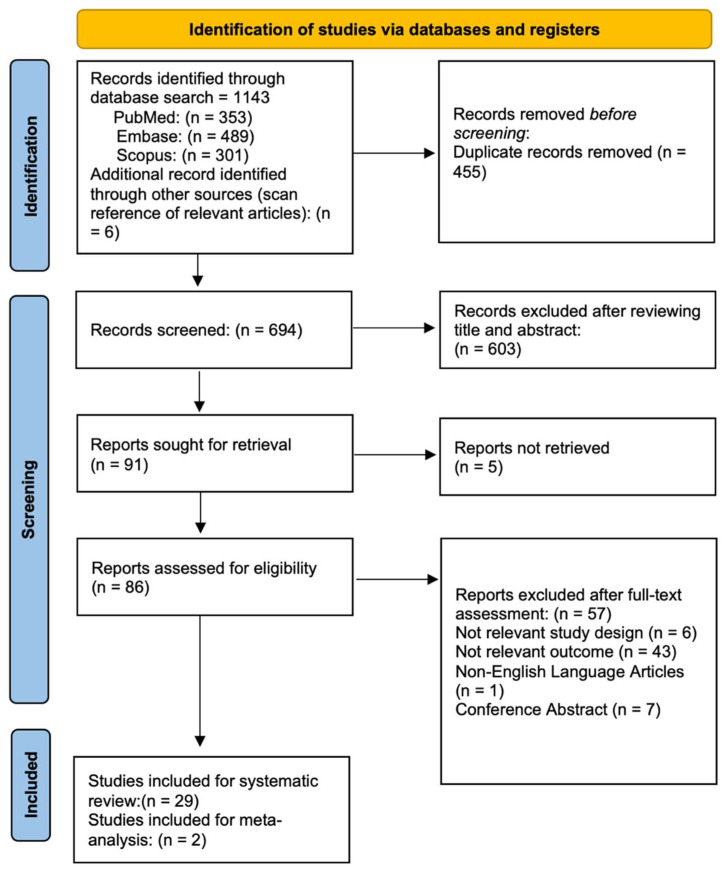
Flow diagram of the study selection process.

The search strategy combined the controlled vocabulary and the keywords according to the indications from each database. The Medical Subject Headings (MeSH) controlled vocabulary were used to search for articles in PubMed, and the Embase Subject Headings (EMTREE) was used in the EMBASE. The keywords were selected based on readings related to the study’s subject. The controlled vocabularies and keywords were used with Boolean operators to extend and direct the search. For addition and restriction, the Boolean operators OR and AND were used. In addition, the investigation was conducted using recognized and extended vocabulary without database filters to achieve a significant sample with a decreased potential loss. Our core search comprised the following terms: “COVID-19” AND “blindness” or relevant synonyms, such as “vision loss”; and “SARS-CoV-2”. In addition, we also hand-searched the bibliographies of included articles to identify further studies that were not found in the initial database search. Figure 1 illustrates a flow diagram of the literature search and screening results.

This review is reported following the Preferred Reporting Items for Systematic Reviews and Meta-Analyses (PRISMA) guidelines. The International Prospective Register of Systematic Reviews (PROSPERO number CRD42022339189, registered on 17 June 2022) has been used to register this systematic review. After the protocol registration, no changes were made. We included the study protocol of the synthesis in Supplementary Material S1. The detailed search strategy and PRISMA Checklist are reported in Supplementary Materials S2 and S3.

### 2.2. Study Selection Data Extraction and Data Synthesis

Articles reporting “vision loss” developed during laboratory-confirmed COVID-19 infection were included.

“Visual loss” was defined according to The International Classification of Diseases 11 (2018), (distance mild visual impairment: visual acuity between <0.5 but ≥0.3 using a decimal scale; distance moderate visual impairment: visual acuity between <0.3 but ≥0.1 using a decimal scale; severe visual impairment: visual acuity between <0.1 but ≥0.05 using a decimal scale; and blindness: visual acuity < 0.5 using a decimal scale or near vision impairment: near visual acuity worse than N6 at 40 cm with existing correction).

All studies reporting patients with neither laboratory-confirmed SARS-CoV-2 infection nor COVID-19-related “visual loss” were excluded. None of the studies reporting “visual loss” developed after COVID-19 infection or vaccination were included. Furthermore, articles were excluded if they were unavailable in the English language or assessed the visual impairment as a prodrome of SARS-CoV-2 infection in otherwise healthy patients. In addition, literature review studies, theses, and dissertations; book chapters; and conference abstracts were not included in our analysis. Reasons for exclusion were documented. In addition, all articles that reported data on “visual loss” cumulative incidence among COVID-19 patients were included in the quantitative analysis.

We contacted the corresponding authors of eligible studies whenever the article could not be retrieved, or we needed to obtain additional information that was not available in the article or online Supplementary Files. Thus, the information was extracted directly from the included studies or provided by the corresponding authors.

Two investigators (M.R. and C.S.) independently extracted baseline and outcome data. If consensus could not be reached, two co-authors (P.A and S.R.) were consulted for adjudication. We extracted the following data from each article: the first author, publication date, country, study design, sample size, study design, average age, gender, visual impairment description, “laterality”, duration between the onset of COVID-19 symptoms and ocular symptoms, comorbidities, number of COVID-19 affected patients and diagnosis. We used Covidence systematic review software© (Veritas Health Innovation, Melbourne, Australia), available at www.covidence.org (accessed on 9 June 2022) [37], to record and evaluate the study data between 22 May 2022 and 09 June 2022.

### 2.3. Risk of Bias Assessment

Two authors (M.R. and C.S.) independently appraised the methodological quality of each cross-sectional and case-report study by using the Newcastle–Ottawa scale (NOS) and the Joanna Briggs Institute (JBI) Critical Appraisal Checklist for Case Reports, which consist of eight yes/no/unclear questions. The JBI critical appraisal checklist for the case series was used for the quality assessment of the case series [38].

Quality assessment data individually appraised by each of the reviewers were compared. If consensus could not be achieved, M.R. and C.S. discussed the discrepancies for adjudication. The data from each reviewer’s quality assessment were compared. M.R. and C.S. discussed the inconsistencies for adjudication if consensus could not be reached.

### 2.4. Statistical Analysis

A random-effects meta-analysis of pooled prevalence and their 95% confidence intervals of COVID-19-affected patients who developed “visual loss” was obtained based on the exact binomial distributions (i.e., number of “events” versus number of “non-events” in a sample) with Freeman–Tukey double-arcsine transformation using the “metaprop” command in STATA (STATA Corp, College Station, TX, USA), version 17.0.

According to Barker et al., a high I^2^ in the context of proportional meta-analysis does not necessarily mean that data are inconsistent, and the results of this test should be interpreted conservatively. Therefore, we did not perform further analysis. Tests to evaluate publication bias, such as Egger’s test and funnel plots, were not performed due to the low number of studies analyzed. Furthermore, Egger’s test and funnel plots were developed in the context of comparative data, and there is no evidence that proportional data adequately adjust for these tests [39]. Statistical significance was determined by a two-sided *p*-value of 0.05.

## 3. Results

### 3.1. Study Selection

Figure 1 illustrates the flow chart of our analysis selection and identification process.

The search yielded 1143 indexed articles (353, 489, and 301 records from PubMed, Embase, and Scopus, respectively). A search of the reference list yielded six other articles. After duplication removal, we screened a total of 694 articles. After the title and abstract screening, we excluded 603 studies, and only 86 full-text studies were retrieved and assessed for final eligibility. Furthermore, 57 articles were excluded because the “visual loss” was developed before or just after the SARS-CoV-2 infection, or COVID-19 was not detected by PCR test. In addition, potential studies were most often excluded due to not fulfilling the study design criteria or being a pre-print and not yet published.

Finally, 29 studies were included in the systematic review [8,9,10,11,12,13,14,15,16,17,18,19,20,21,22,23,24,25,26,27,28,29,30,31,32,33,34,35,36] and two in the meta-analysis [9,23]. The included studies provided data on the number of participants with “visual loss” developed during COVID-19, and the meta-analysis included two studies [9,23] with 288 COVID-19 patients.

### 3.2. Study Characteristics

A summary of the main characteristics including the first author, publication date, country, study design, sample size, study design, average age, gender, visual impairment description, “laterality”, duration between the onset of COVID-19 symptoms and ocular symptoms, comorbidities, number of COVID-19 affected patients and diagnosis are summarized in Table 1. The articles included in this systematic review were published between 28 April 2020 and 22 February 2022. We assessed two cross-sectional studies [9,23], twenty-four case reports [8,10,11,12,13,14,15,16,17,18,19,20,21,22,24,25,27,28,29,30,31,32,33,34,36], and three case series [14,26,35]. The number of participants ranged from one (case reports) to 199 (cross-sectional studies), with 84 out 318 COVID-19 patients experiencing “visual loss”.

**Figure 2:**
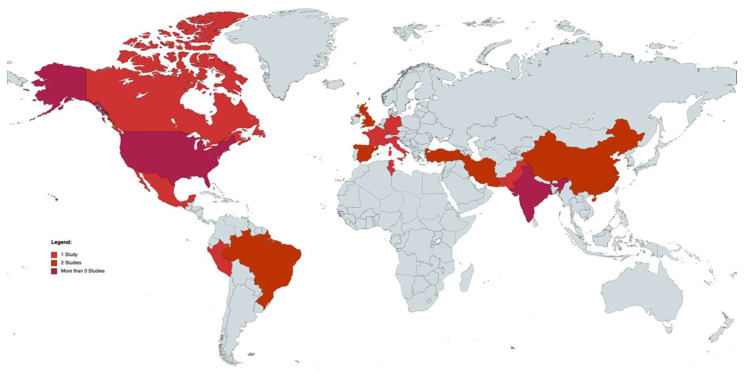
World map of studies included. Map generated through MapChart (MapChart, 2021).

Twenty-seven articles reported the age ((median ± standard deviation), (margin of error)): 46.28 ±3.03 (± 6.55%), n = 31) at the onset of COVID-19 [8,9,10,11,12,13,14,15,16,17,18,19,20,21,22,23,24,25,26,27,28,29,30,31,32,33,35], and twenty-nine articles [8,9,10,11,12,13,14,15,16,17,18,19,20,21,22,23,24,25,26,27,28,29,30,31,32,33,34,35,36] mentioned the sex of the patients, with 175 men and 143 women in total. Overall, seven studies involved patients from the European countries [15,17,21,24,30,35,36], twelve involved patients from the American (north and south) countries [8,13,16,22,23,25,26,28,29,31,33,34], nine involved patients from Asian countries [9,10,11,12,14,18,19,20,27], and one study involved patients from African countries [32] (Figure 2).

### 3.3. ”Visual Loss” Characteristics

Ten Studies reported “visual loss” due to either optic neuritis (ischemic or inflammatory) or optic neuritis alongside panuveitis [8,13,18,21,22,24,29,31,33,36].

Posterior reversible encephalopathy syndrome (PRES) was the leading cause of “visual loss” in two studies [11,15]. Four studies reported “visual loss” as a consequence of a cerebrovascular accident [10,12,19,26], whereas two studies described either central retinal artery occlusion (CRAO) or impending CRAO related to “visual loss” [30,34].

“Visual loss” due to fungal infection (mucormycosis) in corticosteroid-treated patients was described in three case reports and one cross-sectional study [9,14,27,32].

Primary ophthalmic diseases such as paracentral acute middle maculopathy (PAMM), acute viral retinitis, multifocal choroiditis, endophthalmitis, and acute retinal necrosis were the leading “visual loss” causes in five studies [16,17,20,25,28].

Reich et al. and Clarke et al. reported “visual loss” following assisted mechanical ventilation and prone position [24,35].

Where provided, severe visual impairment (visual acuity between <0.1 but ≥0.05 using a decimal scale) and blindness (visual acuity < 0.5 using a decimal scale) incidences were reported.

Visual acuity of no light perception, light perception, hand motion, and counter finger at 4 feet was reported in fifteen studies [10,12,14,15,16,20,24,25,26,29,31,32,33,34,36].

Although bilateral “visual loss” was reported in fourteen studies (18 patients) [9,10,12,13,14,15,18,19,24,25,26,27,29,32], the majority included unilateral “visual loss” (49 patients, 73,13%).

Most studies reported no pre-existing systemic conditions. Pre-existing health conditions including hypercholesterolemia, hypertension, obesity, systemic lupus erythematosus (SLE), and chronic obstructive pulmonary disease were described in thirteen studies [8,9,14,16,17,22,23,24,25,26,27,34,35]. Only one study reported relevant ocular comorbidity, [16] and comorbidities were not described in ten studies [11,12,15,19,21,28,30,31,32,36].

The onset of the “visual loss” following COVID-19 was variable. The included studies reported the onset between one day and four weeks post-onset (average and standard deviation: 9.53 ± 1.60, n = 19, confidence interval ±16.78%). The exact timings of the “visual loss” onset during COVID-19 were not described in eight studies [9,13,18,24,25,27,28,31]. Reich et al. reported neuroretinal damage in three patients after assisted mechanical ventilation [35].

Management of “visual loss” was discussed in a few studies, such as via medication, corticosteroid therapy, and surgical therapy (pars plana vitrectomy for endophthalmitis, endoscopic endonasal debridement, and even evisceration for mucormycosis). Where provided, seven studies reporting the use of corticosteroids revealed improvement [10,18,21,29,30,31,33], and nine studies revealed no improvement in visual function [13,14,15,16,24,25,26,34,36]. Despite the treatment, three studies reported patients’ exitus (two mucormycosis and one stroke-affected patient) [14,26,32].

### 3.4. Meta-Analysis

**Figure 3:**
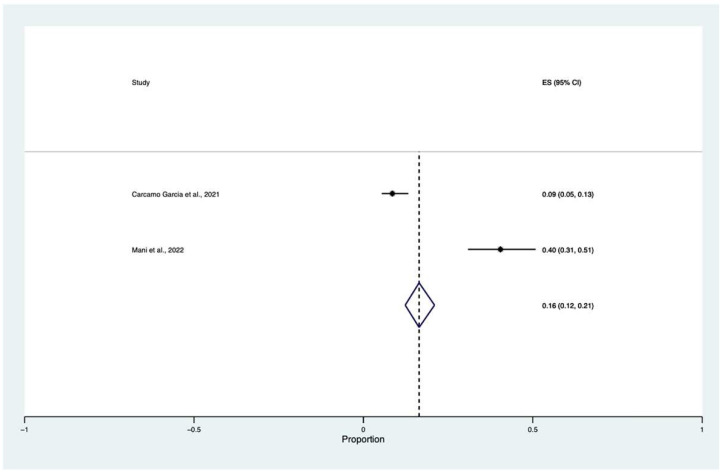
Proportional meta-analysis of cumulative “visual loss” incidence in COVID-19 patients [9,23].

A proportional random meta-analysis was performed to estimate “visual loss” cumulative incidence among COVID-19 patients. The total population was equal to 280, and the sample size varied between 89 and 199. The pooled prevalence of “visual loss” among COVID-19 patients was equal to 0.16 with a confidence interval (CI) between 0.12 and 0.21 (I^2^ = 84.10, z: 12.79, *p* ≤ 0.001) (Figure 3).

### 3.5. Risk of Bias and Publication Bias

Supplementary Material S4 summarizes all studies’ risk of bias evaluation. The quality rating of the cross-sectional studies [9,23] averaged four out of the maximum score on the Newcastle–Ottawa Scale. Overall, the two cross-sectional studies reached a total score of four.

The studies did not report the response rate or the characteristics of the responders and the non-responders. No statistical tests were used to assess the “visual loss” prevalence among the patients. According to the JBI Critical Appraisal Checklist for Case Reports and JBI Critical Appraisal Checklist for Case Series, the quality of the included studies was moderate to good. Most case reports scored 6 out of 8 quality criteria or higher. All case series scored 6 out of 10 quality criteria or higher. Notably, two case series scored 8 out of 10 quality criteria [14,26], whereas one case series scored only six, as it did not provide information regarding follow-up results [35].

## 4. Discussion

Our systematic review and meta-analysis aimed to identify and describe the characteristics of the “visual loss” developed during SARS-CoV-2 infection. Furthermore, we aimed to determine the cumulative incidence of “visual loss” during SARS-CoV-2 infection.

In our meta-analysis, we found that the cumulative incidence of “visual loss” is 16% (CIs: 0.12–0.21) in confirmed cases of COVID-19. This number should be interpreted with precaution because of the low level of evidence (one study provided data of “visual loss” in patients with concomitant COVID-19 and rhino-orbital-mucormycosis) and high heterogeneity between the papers [9].

Among the sensory impairment symptoms during COVID-19, “visual loss” is less frequently reported than olfactory and gustatory changes. Indeed, few reports reporting “visual loss” during SARS-CoV-2 infection have been published since the pandemic outbreak. Despite many papers reporting several ocular symptoms such as conjunctivitis, epiphora, pain, and redness, few articles report “vision loss” [40].

Many patients experiencing “vision loss” underwent an ischemic or inflammatory optic neuropathy, mucormycosis, uveitis, or cerebrovascular accidents.

COVID-19 infection has been associated with prothrombotic effects due to virus-induced cytokine storm that can activate and upregulate the coagulation, triggering the formation of a thrombus that may lead to ophthalmic artery occlusion (CRAO), central retinal artery occlusion, central retinal vein occlusion, ischemic optic neuropathy, occipital cortical infarct, or acute macular neuroretinopathy [41]. Furthermore, SARS-CoV-2 may engage the endothelium, increasing the permeability of the blood–brain barrier and leading to encephalopathy, encephalitis, and thrombosis [42].

Murchinson et al. first reported CRAO as the initial manifestation of COVID-19 in a patient with an unremarkable neurologic exam [34]. Cyr et al. reported two cases of COVID-19-positive patients with severe bilateral “vision loss” due to an acute bilateral occipital territorial ischemic infarct in a 61-year-old patient with a seven-day history of COVID-19-like symptoms and a chronic infarction in the right temporal-parietal lobe and bilateral medial occipital lobes in a 34-year-old woman with a history of systemic lupus erythematosus [26]. Khan et al. first described a bilateral occipital stroke leading to bilateral “vision loss” in a 60-year-old man with no previous risk factors for the cerebrovascular incident [19].

Furthermore, the virus-induced cytokine storm leads to a systemic inflammatory state, causing endothelial dysfunction and determining vascular leakage and edema formation, and endothelial activation resulting in the release of the immunogenic and vasoactive substance [43]. Kaya et al. and Elhassan et al. described a bilateral reversible cortical blindness and Anton’s cortical blindness in patients affected by posterior reversible leukoencephalopathy with modest blood pressure fluctuations and no hypertension history [11,15].

The leading cause of visual loss across the studies was optic neuropathy. SARS-CoV-2 can affect the nervous system through different routes. It can enter the nervous system hematogenously by infecting the choroid plexus or meninges or spreading through the olfactory nerves. Moreover, a mechanism of molecular mimicry in which viral antigens would induce an immune response against self-proteins may be responsible for tissue injury [44]. Zhou et al. illustrated a case of SARS-CoV-2 infection followed by myelin oligodendrocyte glycoprotein (MOG)-IgG-related optic neuritis and myelitis that strengthened the immune-based pathogenesis. Furthermore, the upregulated coagulation may raise small capillary ischemic events, leading to ischemic optic neuritis [29].

Clarke et al. first described a case of non-arteritic ischaemic optic neuropathy after mechanical ventilation in the prone position. Indeed, prone positioning might alter ocular hemodynamics, raising intraocular pressure (IOP) and thus reducing optic nerve perfusion. The patient underwent eight episodes of prone position during mechanical ventilation to treat COVID-19-related acute respiratory distress syndrome (ARDS), and just after awakening, he reported bilateral “vision loss” [24]. Reich et al. described three cases of “visual loss” following assisted mechanical ventilation. Fundus examination revealed ischemic lesions of the retina, atrophy of inner retinal layers, and optic atrophy [35].

Benito Pascual et al. described a case of panuveitis and optic neuritis preceded by conjunctivitis prior to the onset of pulmonary symptoms [21]. Subsequently, Liu et al. described acute viral retinitis and optic neuritis followed by cataract and glaucoma due to COVID-19 infection. This inflammation might be due either to direct infiltration of the virus via ACE2 or an intraocular autoimmune response [20].

COVID-19 may lead to various opportunistic infections. Indeed, the altered immune response and the use of corticosteroids may increase the risk of superadded infections after a prolonged period in intensive care units. During COVID-19, many cytokines such as IL-6, IL-10, and TNF-α are markedly higher, whereas T lymphocytes are much lower. In patients with predisposing factors, COVID-19 may raise superinfections [9]. Reactivation of Herpes simplex virus and mucormycosis infection during COVID-19 were described as potential conditions that determine “visual loss”. COVID-19 direct injury to human islet cells, determining beta cell damage and the endogenous insulin secretion’s reduction, as well as the cytokine storm, lead to insulin resistance. In addition, commonly used drugs such as glucocorticoids and remdesivir further alter the glucose homeostasis, predisposing the patient to opportunistic infections. Notably, fungal infections such as mucormycosis may be promoted by ketoacidosis-induced free-iron availability [9].

Mani et al. reported that 19% of patients experienced “visual loss” due to mucormycosis [9], whereas Crane et al. described a case of Klebsiella endophthalmitis in a patient with multiple comorbidities such as liver cirrhosis, diabetes, and emphysematous prostatitis [25]. Moreover, Gonzalez et al. described a reactivation of HSV causing acute retinal necrosis in a patient with a prior history of necrotizing herpetic retinitis in the fellow eye [16].

This study has several limitations. First, the meta-analysis involved only two studies with different sample sizes. Furthermore, one study only evaluated the “visual loss” in ROMC-COVID-19-affected patients. Thus, the pooled cumulative incidence should be interpreted cautiously, and our findings should be interpreted while keeping in mind this significant limitation. Second, we could not evaluate the follow-up, as many studies lack this critical data. Third, the majority of the studies were subjected to a qualitative analysis. Fourth, the cross-sectional studies scored four out of the maximum score on the Newcastle-Ottawa Scale. However, to the best of our knowledge, this is the first study that deeply analyzes the association between COVID-19 and “visual loss”. In addition, many systematically included studies involving a high number of patients from different countries make our findings generalizable and represent one of our study’s strengths. Nonetheless, future research that aims to prevent any COVID-19-related blindness disease should be further conducted. Studies with larger sample sizes are needed to further investigate the pooled cumulative incidence of “visual loss” during SARS-CoV-2 infection.

## 5. Conclusions

“Visual loss” during SARS-CoV-2 infection is a rare finding. Despite the low incidence, many cases have been reported in the literature. Indeed, COVID-19 might cause “visual loss” through several mechanisms. Therefore, COVID-19 should be considered in patients who have recently developed “visual loss”, and clinicians should be aware of this uncommon event to avoid blindness in everyday clinical practice.

## Figures and Tables

**Table 1:** Characteristics of studies included in the systematic review.

Study Name	Country	Publication Date	Sample Size Study Design Age (Media ± SD) Gender	Visual Impairment Description	“Laterality”	Visual Acuity	Duration between the Onset of COVID-19 Symptoms and Ocular Symptoms	Comorbidities	Patients with COVID-19 (N and %)	Diagnosis
Kaya et al. [11]	Turkey	28 April 2020	N = 1 Case report 38 Male	Vision loss in both eyes	Bilateral	OU: perception light	5 days	NA	1	PRES
Selvaraj et al. [8]	USA	10 June 2020	N = 1 Case Report 50 Female	Acute, painless RE monocular visual disturbance, described as a white cloud and blurriness involving most of her RE, sparing the superior nasal aspect.	Monocular	RE: 20/70	7 days	NA	1	PION
Reich et al. [35]	Germany	17 August 2020	N = 1 each case Case sries Case 1: 64 Case 2: 43 Case 3: 60 Case 1: Female Case 2 and 3: Male	Immediately after regaining full consciousness, the patients reported visual impairment.	Case 1 and 3: Bilateral Case 2: Monolateral	Case 1: OU: 0.5 LogMar Case 2: LE: 1 LogMar Case 3: RE: light perception LE: 1.0 LogMar	Soon after extubation (after 7–16 days of mechanical ventilation)	Case 1: obesity Case 2: arterial hypertension and medical history of carbon monoxide poisoning Case 3: arterial hypertension, insulin-dependent diabetes mellitus, grade 1 obesity, and nicotine abuse	1 each case	Case 1: Optic Atrophy Case 2: High Intraocular Pressure (IOP > 50) Case 3: Optic Atrophy
Cyr et al. [26]	USA	September 2020	N = 1 each case Case series Patient 1: 61 Patient 2: 34 patient 1: male patient 2: Female	Patient 1: sudden, painless loss of vision for 2 days Patient 2: sudden, painless loss of vision of two-day duration.	Patient 1: bilateral Patient 2: bilateral	Patient 1: light perception Patient 2: light perception	Patient 1: 7 days Patient 2: 10 days	Patient 1: non–insulin-dependent diabetes mellitus Patient 2: four systemic lupus erythematosus, hypertension, end-stage renal disease on hemodialysis, chronic obstructive pulmonary disease	1 each case	Patient 1: acute bilateral occipital territorial ischemic infarct Patient 2: acute infarct in the right frontal lobe, acute left posterior temporal-occipital territorial infarction and bilateral medial occipital
Zhou et al. [29]	USA	September 2020	N = 1 Case report 26 Male	Bilateral, subacute, sequential vision loss first affecting the LE, then the RE 3 days later	Bilateral	RE: HM LE: 20/250	“Few days”	None	1	Severe optic neuritis and myelitis
Benito Pascual et al. [21]	Spain	1 September 2020	N = 1 Case Report 60 Female	Ocular pain, blurred vision, and redness in her LE	Monolateral	LE: 20/200	14 days	NA	1	Panuveitis and Optic Neuritis
Khan et al. [19]	Pakistan	3 September 2020	N = 1 Case report 60 Male	Bilateral visual loss	Bilateral	NA	24 h	NA	1	Cortical blindness secondary to occipital lobe stroke
Invernizzi et al. [30]	Italy	25 September 2020	N = 1 Case report 54 Female	Scotomas and decreased vision in her RE	Monolateral	RE: 20/40	10 days	NA	1	Impending Central Retinal Vein Occlusion
Gascon et al. [17]	France	06 Oct 2020	N = 1 Case Report 50 Male	LE: negative scotoma and acute onset of dyschromatopsia and decreased visual acuity	Monolateral	LE: 20/63	8 days	Splenectomy and RE: Glaucoma	1	Acute macular neuroretinopathy and paracentral acute middle maculopathy
Catharino et al. [22]	Brazil	18 November 2020	N = 1 Case Report 64 Male	NA	Monolateral	NA	The same day	hypertension	1	Optic Neuritis
Murchison et al. [34]	USA	15 December 2020	N = 1 Case Report “Fifth decade” Male	RE: Acute onset of painless visual loss	Monolateral	RE: HM	3-weeks	Hypertension, tobacco use, and occasional marijuana use	1	CRAO
Francois et al. [36]	France	17 December 2020	N = 1 Case Report “Late 50 s” Female	Blurred vision and redness in her RE and temporary (eight-day history) pain when mobilizing the globe	Monolateral	RE: HM	2 days	NA	1	Ocular neuropathy and panuveitis
Elhassan 2021 [15]	UK	19 January 2021	N = 1 Case Report 52 Female	Complete cortical blindness with poor insight into the extent of her visual impairment, often claiming to be able to see (Anton’s syndrome) and hallucinations	Bilateral	No perception light	31	None	1	PRES
Liu et al. [20]	China	03 February 2021	N = 1 Case Report 66 Female	“Shadows similar to cotton wool” with her LE followed by monocular blindness	Monolateral	No light perception	First symptoms 10 days after COVID-19, blindness after 3 weeks from initial symptoms	None	1	Acute viral retinitis, optic neuritis, uveitis and secondary glaucoma
De Souza et al. [28]	Brazil	19 February 2021	N = 1 Case Report 23 Male	Acute painless loss of central vision in his RE	Monolateral	RE: 20/800	NA	NA	1	Multifocal choroiditis
Katti et al. [10]	India	16 March 2021	N = 1 Case Report 66 Male	Sudden bilateral loss of vision	Bilateral	RE/LE: no light perception	10 days	None	1	Pituitary macroadenoma with apoplexy and stroke
Rodríguez-Rodríguez et al. [13]	Mexico	23 March 2021	N = 1 Case Report 55 Female	Unilateral, gradual visual loss, decreasing visual acuity, and chromatic impairment	Bilateral	RE: 20/40 LE: 20/200	NA	None	1	Optic neuritis
Veisi et al. [14]	Iran	10 April 2021	N = 1 each case Case Series Case 1: 40 Case 2: 54 Case 1: Female Case 2: Male	Case 1: bilateral visual loss and complete ophthalmoplegia of the RE Case 2: vision loss, proptosis, orbital inflammation, and complete ophthalmoplegia on the left side	Case 1: bilateral Case 2: monolateral	Case 1: no light perception Case 2: LE light percepetion	Case 1: 15 days Case 2: 7 days	Case 1 None Case 2 Non-insulin-dependent diabetes mellitus	1 per case	Case 1: Mucormycosis Case 2: Rhino-orbital mucormycosis
Carcamo Garcia et al. [23]	Perù	14 April 2021	N = 199 Cross-sectional study 42.8 ±15.1 85 males and 114 females	Visual changes 24 (12%): Visual symptoms: 23 (11.6%) Unilateral 0 (0%) Bilateral 15 (65.2%) Deficient color vision 4 (17.4%) Vision loss 17 (73.9%) Double vision 3 (13%)	NA	NA	8 ± 6 days	Hypercholesterolemia (12%), followed by hypertension (10%), prior history of tuberculosis or other pulmonary disease (9%) and diabetes (7%), cancer (4%); chronic kidney disease (2%) cerebrovascular disease or stroke (1%). Nearly 10% of the cohort had a history of smoking or were current smokers.	199	Bilateral visual changes and decreased visual acuity were the most common symptoms in patients with mild-moderate COVID-19 infection.
Crane et al. [25]	USA	21 April 2021	N = 1 Case Report 35 Male	Vision loss with no associated pain or redness started in the LE but very quickly involving the RE	Bilateral	RE/LE: Light perception	NA	diabetes, cirrhosis	1	Endogenous Klebsiella endophthalmitis.
Deane et al. [31]	USA	13 June 2021	N = 1 Case Report 21 Female	Blurry vision in her LE associated with one-week history of severe headaches with pain with movements in all directions in her LE	Monolateral	LE: Hand Motion	NA	NA	1	Optic Neuritis
Eswaran et al. [27]	India	13 June 2021	N = 1 Case Report 31 Male	Bilateral proptosis, loss of vision and ophthalmoplegia	Bilateral	NA	NA	Diabetes	1	Mucormycosis
Clarke et al. [24]	UK	13 July 2021	N = 1 Case Report 55 Male	Profound bilateral vision loss after cessation of sedation	Bilateral	LE: 3/30 unaided RE: counting fingers	NA	Hypercholesterolemia and hypertension	1	NAION due to Prone Position
Gonzalez et al. [16]	USA	19 July 2021	N = 1 Case Report 32 Female	Sudden vision loss in her LE, associated with a one-week history of pain, redness, and photophobia	Monocular	LE: perception light	24 h	Left retinal detachment secondary to necrotizing herpetic retinitis	1	Acute retinal necrosis
Atum et al. [12]	Turkey	23 July 2021	N = 1 Case Report 84 Male	Sudden vision loss	Bilateral	HM	5 days	NA	1	Bilateral occipital ischemic stroke
Micieli et al. [33]	Canada	29 July 2021	N = 1 Case Report 31 Male	RE vision loss after 10-day history of pain that worsened with eye movements and blurred vision	Monolateral	RE: CF at 4 feet	12	None	1	Optic Neuritis
Eslamiyeh et al. [18]	Iran	8 August 2021	N = 1 Case Report 8 Male	Sudden bilateral and progressive blurring of vision in the RE	Bilateral	RE: 2/10 LE: 4/10	NA	NA	1	Optic Neuritis
Malek et al. [32]	Tunisie	18 October 2021	N = 1 Case Report 20 Male	Rapid bilateral visual loss with left periorbital pain, proptosis, palpebral edema, and swelling	Bilateral	No LP	7 days	NA	1	Rhino-orbito-cerebral mucormycosis, left cavernous sinus and internal carotid thrombosis together with a right CRAO
Mani et al. [9]	India	25 February 2022	N = 89 Cross-sectional study 54.71 ± 11.03 70 males and 19 females	ROCM (stage 3c): 1 patient: Bilateral orbital involvement with loss of vision ROCM (stage 3d) 35 patients: central retinal artery occlusion or involvement of orbital apex, superior orbital fissure, inferior orbital fissure with loss of vision	One patient bilateral, 35 patients monolateral	NA	NA	Diabetes	89	Rhino-orbital-cerebral mucormycosis

Abbreviations: NA: not applicable; ROMC: rhino-orbital-mucormycosis; CRAO: central retinal artery occlusion; RE: right eye; LE: left eye; OU: oculus uterque; N: number; CF: counter fingers; HM: hand motions; USA: United States of America, UK: United Kingdom; NAION: non-arteritic anterior ischemic optic neuropathy; PCR: polimerase chain reaction; IOP: Intraocular pressure, PION: posterior ischemic optic neuropathy; PRES: posterior reversible encephalopathy syndrome.

## Data Availability

Not applicable.

## Supplementary Materials

The following supporting information can be downloaded at: https://www.mdpi.com/article/10.3390/vision6040060/s1, Supplementary Material S1: Protocol; Supplementary Material S2: Detailed Search Strategy; Supplementary Material S3: PRISMA Checklist [45]; Supplementary Material S4: risk of bias evaluation.
