# Supplementary material for: “Vision Loss” and COVID-19 Infection: A Systematic Review and Meta-Analysis
Source: Vision (Basel). 2022 Sep 23;6(4):60. doi: 10.3390/vision6040060 (PMC9589937; doi:10.3390/vision6040060)
Supplement: Supplementary file 1 [file vision-06-00060-s001.zip › Supplementary Material S1.pdf]

Supplementary Material S1: Protocol

"Vision Loss" and COVID-19 infection: A Systematic Review and Meta-Analysis"

Matteo Ripa, MD [ab]; Lorenzo Motta MD [c], PhD; Chiara Schipa, MD [bd]; Stanislao Rizzo MD [abe]; Liliana Sollazzi MD [bd]; Paola Aceto, MD, PhD [bd]

a. Ophthalmology Unit, "Fondazione Policlinico Universitario A. Gemelli IRCCS", Rome, Italy

b. Catholic University "Sacro Cuore", Rome, Italy

c. Department of Ophthalmology, William Harvey Hospital, East Kent Hospitals University NHS Foundation Trust, Ashford TN24 0LZ, UK

d.Department of Emergency, anesthesiological and reanimation sciences, Fondazione Policlinico Universitario A. Gemelli IRCCS, Rome, Italy

e. Consiglio Nazionale delle Ricerche, Istituto di Neuroscienze, Pisa, Italy

Correspondence: Chiara Schipa, MD, Department of Emergency, anaesthesiological and reanimation sciences, "Fondazione Policlinico Universitario A. Gemelli IRCCS", Catholic University "Sacro Cuore", Rome, Italy Largo A Gemelli, 8 - 00168 Rome, Italy.

Email: chiara.schipa13@gmail.com Telephone number: +393290730977

ORCID iD: Chiara Schipa https://orcid.org/0000-0003-3856-5616

This study has been registered in PROSPERO (CRD42022339189). Deviations will be specified in the published manuscript for this systematic review and meta-analysis. This work is investigator-initiated and is not pending any specific funding.

Introduction

Severe acute respiratory syndrome coronavirus-2 (SARS-CoV-2) is a single-stranded RNA virus that belongs to the Coronaviridae family. [1]

The World Health Organization (WHO) declared the coronavirus disease 2019 (COVID-19) outbreak a global pandemic on March 11, 2020, which led to a significant economic and healthcare burden. [2]

Current available diagnostic tests to detect COVID-19 include a triad of complementary approaches. The Polymerase chain reaction (PCR) is the most highly sensitive and specific molecular test to detect SARS-CoV-2 nucleic acids presence representing the gold standard technique because of its sensitivity and specificity. It uses primers matching a segment of the SARS-CoV-2 genetic material to detect COVID-19. [3]

After the exposure, the average incubation period may range from four to five days. [4] A wide range of symptoms has been associated with the SARS-CoV-2 infection, whose severity may vary from being asymptomatic to death. Although most patients either remain asymptomatic or experience common viral infection symptoms such as fever, cough, and fatigue, some patients may develop atypical symptoms such as neurological (headaches, loss of taste, smell), and ophthalmological symptoms (conjunctivitis, epiphora, and vision loss).[5]

Based on multiple cross-sectional studies, the incidence of ocular manifestation in COVID-19 patients might be as high as 30%.[6] At the pandemic beginning, many physicians reported eye redness and irritation in patients, describing 'conjunctival congestion' in Wuhan, China. In a recent systematic review and meta-analysis, Inomata et al. reported clinical and prodromal ocular symptoms in patients with COVID-19. The most common ocular findings among COVID-19 patients were conjunctivitis (86.4%), ocular pain (34.4%), dry eye (33.3%), and floaters (6.7%). [7]

"Visual loss" in COVID-19 patients was reported in a few articles. Nonetheless, it has

been observed that its onset may be due to viral neurotropism and indirect immunologic and neurovascular effects.

Furthermore, despite extensive research on sensory manifestations of COVID-19 since the start of the pandemic, only a few articles and no meta-analysis paper have assessed "vision loss" as a symptom during the SARS-CoV-2 infection. [8–36]

The present paper intends to systematically review current evidence regarding visual loss, caused by SARS-CoV-2, as well as determine the cumulative incidence of this symptom through a meta-analysis. In addition, we further aim to identify the characteristics of the "visual loss", thus evaluating factors that could contribute to understanding the association between COVID-19 and "visual loss".

Methods

This protocol was written with reference to the PRISMA (Preferred Reporting Items for Systematic Reviews and Meta-Analyses) guidelines. Ethical approval is not required as we will only conduct a secondary analysis of available data published in the literature. Our results will be submitted for peer-review publication and/or conference presentations.

Eligibility Criteria

Inclusion criteria:

1. Population: patients with "vision loss" developed during laboratory-confirmed Covid-19 infection.
2. Outcomes: cumulative incidence of "visual loss" in Covid-19 patients.
3. Study type: No studies restriction

Exclusion criteria:

1. Article not published in English language

2. All studies reporting patients with neither laboratory-confirmed Covid-19 infection nor Covid-19-related visual loss or studies reporting visual loss devolped after covid infection or vaccination.
3.

Information Sources

We will systematically search 3 databases (PubMed, Embase and Scopus). We will also hand-search the reference lists of the included articles and relevant reviews or journals, where applicable. We will attempt contact with the corresponding authors to obtain additional unpublished information.

Search Strategy

PubMed

Free Text
No limits applied

#	Search Term	No. Results
1	"vision"	
2	"loss"	
3	1 AND 2	
4	"blindness"	
5	3 OR 4	
6	" COVID-19" OR "SARS-CoV-2"	
7	5 AND 6	

Controlled Vocabulary: Medical Subject Headings (MeSH)
No limits applied

#	Search Term	No. of Results
1	("Vision Loss"[MeSH]) AND "COVID-19"[MeSH]	
2	("Blindness"[MeSH]) AND "COVID-19"[MeSH]	
3	("Loss of vision"[MeSH]) AND " COVID-19"[MeSH]	
4	("Poor Vision"[MeSH]) AND " COVID-19"[MeSH]	
5	("low vision"[MeSH]) AND " COVID-19"[MeSH]	

All MeSH search results had already been found in the free-text search.

Embase

Free Text
[embase]/lim, no other limits applied

Mapping options enabled:

- map to preferred term in Emtree
- search also as free text in all fields
- explode using narrower Emtree terms

search as broadly as possible

#	Search Term	No. of Results
1	(("vision") AND ("loss) OR ("blindness") AND (" COVID-19" OR "SARS-CoV-2")	

Scopus

Free Text

#	Search Term	No. of Results
1	TITLE-ABS-KEY("vision") AND TITLE-ABS-KEY ("loss") OR (TITLE-ABS-KEY (("blindness")) AND TITLE-ABS-KEY ("COVID-19" OR"SARS-CoV-2")	

Data Management

We will export the search results to Covidence systematic review software© (Veritas Health Innovation, Melbourne, Australia) available at www.covidence.org [37] to remove duplicates and manually screenthe records.

Selection Process

We will screen potentially eligible studies based on title and abstract, following which, wewill retrieve full texts for evaluation. This will be done by 2 independent reviewers.

Data Extraction & Data Items

We will extract the following data from each article: the first author, publication date, country, study design, sample size, study design, average age, gender, visual impairment description, "laterality", duration between the onset of COVID-19 symptoms and ocular symptoms, comorbidities, number of Covid-19 affected patients and diagnosis-

Two investigators will independently extract baseline and outcome data. If consensus is not be reached, two co-authors will be consulted for adjudication. Reasons for exclusion will be documented.

Risk of Bias

We plan to use the Newcastle-Ottawa Scale (NOS), to evaluate the risk of bias at the outcome level. As per the NOS grading in past reviews, we will grade studies as having a high (<5 stars), moderate (5-7 stars) or low risk of bias (≥8 star). For longitudinal studies, the NOS assigns a score out of 9 to each study (where 9 indicates that the study meets all9 criteria for quality assessments and 0 indicates that the study does not meet any of the criteria) based on potential domains of bias, such as selection, comparability, and outcome. For cross-sectional studies, the modified NOS assigns a score out of 10 to each study. Moreover, the Joanna Briggs Institute (JBI) Critical Appraisal Checklist for Case Reports which consist of eight yes/no/unclear questions and the JBI critical appraisal checklist for case series was used for quality assessment of the case series will be used. [38]

Statistical Analysis

A random-effects meta-analysis of pooled prevalence and their 95% confidence intervals of Covid-19 affected patients who developed "visual loss" will be used based on the exact binomial distributions (i.e. number of "events" versus number of

"non-events" in a sample) with Freeman-Tukey double-arcsine transformation using the "metaprop" command in STATA (STATA corp, USA), version 17.0.

Keywords

Covid-19; visual impairment, poor vision

References

1. Nabil, A.; Uto, K.; Elshemy, M.M.; Soliman, R.; Hassan, A.A.; Ebara, M.; Shiha, G. Current Coronavirus (SARS-CoV-2) Epidemiological, Diagnostic and Therapeutic Approaches: An Updated Review until June 2020. *EXCLI J* **2020**, *19*, 992–1016, doi:10.17179/EXCLI2020-2554.
2. Savastano, A.; Ripa, M.; Savastano, M.C.; Kilian, R.; Marchini, G.; Rizzo, S. Impact of the COVID-19 Pandemic on Ophthalmologic Outpatient Care: Experience from an Italian Tertiary Medical Center., doi:10.1080/07853890.2021.1964036.
3. Eastin, C.; Eastin, T. Clinical Characteristics of Coronavirus Disease 2019 in China: Guan W, Ni Z, Hu Y, et al. N Engl J Med. 2020 Feb 28 [Online Ahead of Print] DOI: 10.1056/NEJMoa2002032. *The Journal of Emergency Medicine* **2020**, *58*, 711, doi:10.1016/J.JEMERMED.2020.04.004.
4. Gandhi, M.; Yokoe, D.S.; Havlir, D. v. Asymptomatic Transmission, the Achilles' Heel of Current Strategies to Control Covid-19. *New England Journal of Medicine* **2020**, *382*, 2158–2160, doi:10.1056/NEJME2009758/SUPPL_FILE/NEJME2009758_DISCLOSURES.PDF.
5. Guan, W.; Ni, Z.; Hu, Y.; Liang, W.; Ou, C.; He, J.; Liu, L.; Shan, H.; Lei, C.; Hui, D.S.C.; et al. Clinical Characteristics of Coronavirus Disease 2019 in China. *N Engl J Med* **2020**, *382*, 1708–1720, doi:10.1056/NEJMOA2002032.
6. Wu, P.; Duan, F.; Luo, C.; Liu, Q.; Qu, X.; Liang, L.; Wu, K. Characteristics of Ocular Findings of Patients With Coronavirus Disease 2019 (COVID-19) in Hubei Province, China. *JAMA Ophthalmol* **2020**, *138*, 575–578, doi:10.1001/JAMAOPHTHALMOL.2020.1291.
7. Inomata, T.; Kitazawa, K.; Kuno, T.; Sung, J.; Nakamura, M.; Iwagami, M.; Takagi, H.; Midorikawa-Inomata, A.; Zhu, J.; Fujimoto, K.; et al. Clinical and Prodromal Ocular Symptoms in Coronavirus Disease: A Systematic Review and Meta-Analysis. *Invest Ophthalmol Vis Sci* **2020**, *61*, doi:10.1167/IOVS.61.10.29.
8. François, J.; Collery, A.S.; Hayek, G.; Sot, M.; Zaidi, M.; Lhuillier, L.; Perone, J.M. Coronavirus Disease 2019–Associated Ocular Neuropathy With Panuveitis: A Case Report. *JAMA Ophthalmology* **2021**, *139*, 247–249, doi:10.1001/JAMAOPHTHALMOL.2020.5695.
9. Selvaraj, V.; Sacchetti, D.; Finn, A.; Kwame Dapaah-Afriyie, ; Acute Vision Loss in a Patient with COVID-19.

10. Mani, S.; Thirunavukkarasu, A. A Clinico-Pathological Study of COVID-19 Associated Rhino-Orbital-Cerebral Mucormycosis. *Indian J Ophthalmol* **2022**, *70*, 1013–1018, doi:10.4103/IJO.IJO_2366_21.
11. Katti, V.; Ramamurthy, L.; Kanakpur, S.; Shet, S.; Dhoot, M. Neuro-Ophthalmic Presentation of COVID-19 Disease: A Case Report. *Indian J Ophthalmol* **2021**, *69*, 992–994, doi:10.4103/IJO.IJO_3321_20.
12. Kaya, Y.; Kara, S.; Akinci, C.; Kocaman, A.S. Transient Cortical Blindness in COVID-19 Pneumonia; a PRES-like Syndrome: Case Report. *J Neurol Sci* **2020**, *413*, doi:10.1016/J.JNS.2020.116858.
13. Atum, M.; Demiryüre, B.E. Sudden Bilateral Vision Loss in a COVID-19 Patient: A Case Report. *Indian J Ophthalmol* **2021**, *69*, 2227–2228, doi:10.4103/IJO.IJO_3706_20.
14. Rodríguez-Rodríguez, M.S.; Romero-Castro, R.M.; Alvarado-de la Barrera, C.; González-Cannata, M.G.; García-Morales, A.K.; Ávila-Ríos, S. Optic Neuritis Following SARS-CoV-2 Infection. *Journal of NeuroVirology* **2021**, *27*, 359–363, doi:10.1007/s13365-021-00959-z.
15. Veisi, A.; Bagheri, A.; Eshaghi, M.; Rikhtehgar, M.H.; Rezaei Kanavi, M.; Farjad, R. Rhino-Orbital Mucormycosis during Steroid Therapy in COVID-19 Patients: A Case Report. *European Journal of Ophthalmology* **2021**, doi:10.1177/11206721211009450.
16. Elhassan, M.; Saidahmed, O.; Adebayo, A.; Archibald, N. Persistent Cortical Blindness Following Posterior Reversible Encephalopathy Syndrome (PRES) as a Complication of COVID-19 Pneumonia. *Cureus* **2021**, *13*, 1–5, doi:10.7759/cureus.12794.
17. Gonzalez, M.P.; Rios, R.; Pappaterra, M.; Hernandez, M.; Toledo, A.; Santos, C.; Emanuelli, A.; Kurup, S.K.; Oliver, A.L. Reactivation of Acute Retinal Necrosis Following SARS-CoV-2 Infection. *Case Reports in Ophthalmological Medicine* **2021**, *2021*, 1–4, doi:10.1155/2021/7336488.
18. Gascon, P.; Briantais, A.; Bertrand, E.; Ramtohul, P.; Comet, A.; Beylerian, M.; Sauvan, L.; Swiader, L.; Durand, J.M.; Denis, D. Covid-19-Associated Retinopathy: A Case Report. *Ocular Immunology and Inflammation* **2020**, *28*, 1293–1297, doi:10.1080/09273948.2020.1825751.
19. Eslamiyeh, H.; Jafari, M. Binocular Optic Neuritis in an Eight-Year-Old Boy Due to Covid-19 Infection. *Iranian Journal of Pediatrics* **2021**, *31*, 0–4, doi:10.5812/ijp.111798.
20. Khan, A.W.; Ullah, I.; Khan, K.S. Ischemic Stroke Leading to Bilateral Vision Loss in COVID-19 Patient—A Rare Case Report. *Journal of Medical Virology* **2021**, *93*, 683–685, doi:10.1002/jmv.26484.
21. Liu, L.; Cai, D.; Huang, X.; Shen, Y. COVID-2019 Associated with Acquired Monocular Blindness. *Current Eye Research* **2021**, *46*, 1247–1250, doi:10.1080/02713683.2021.1874027.
22. Benito-Pascual, B.; Gegúndez, J.A.; Díaz-Valle, D.; Arriola-Villalobos, P.; Carreño, E.; Culebras, E.; Rodríguez-Avial, I.; Benitez-Del-Castillo, J.M. Panuveitis and

Optic Neuritis as a Possible Initial Presentation of the Novel Coronavirus Disease 2019 (COVID-19). *Ocular Immunology and Inflammation* **2020**, *28*, 922–925, doi:10.1080/09273948.2020.1792512.
23. Catharino, A.M. da S.; Neves, M.A.O.; Nunes, N. dos S.M.; do Nascimento, J.S.F.; do Nascimento, J.K.F.; Castro, R.R.T.; Azizi, M.A.A.; Airão, A.R.; Alvarenga, R.M.P. COVID-19 Related Optic Neuritis: Case Report. *Journal of Clinical Neurology and Neuroscience* **2020**, doi:10.52588/clinical-neurology-neuroscience.1.10.
24. Carcamo Garcia, M.H.; Garcia Choza, D.D.; Salazar Linares, B.J.; Diaz, M.M. Neurological Manifestations of Patients with Mild-to-Moderate COVID-19 Attending a Public Hospital in Lima, Peru. *eNeurologicalSci* **2021**, *23*, 100338, doi:10.1016/j.ensci.2021.100338.
25. Clarke, K.M.; Riga, V.; Shirodkar, A. lee; Meyer, J. Proning Related Bilateral Anterior Ischaemic Optic Neuropathy in a Patient with COVID-19 Related Acute Respiratory Distress Syndrome. *BMC Ophthalmology* **2021**, *21*, 1–6, doi:10.1186/s12886-021-02028-9.
26. Crane, A.B.; Abreu Diaz, M.C.; Jiang, Y.; Pergament, K.M. Rare Case of Endogenous Klebsiella Endophthalmitis Associated with Emphysematous Prostatitis in a Patient with Diabetes, Cirrhosis and COVID-19. *BMJ Case Reports* **2021**, *14*, 1–4, doi:10.1136/bcr-2020-240425.
27. Cyr, D.G.; Vicidomini, C.M.; Siu, N.Y.; Elmann, S.E. Severe Bilateral Vision Loss in 2 Patients With Coronavirus Disease 2019. *J Neuroophthalmol* **2020**, *40*, 403–405, doi:10.1097/WNO.0000000000001039.
28. Eswaran, S.; Balan, S.K.; Saravanam, P.K. Acute Fulminant Mucormycosis Triggered by Covid 19 Infection in a Young Patient. *Indian Journal of Otolaryngology and Head and Neck Surgery* **2021**, doi:10.1007/s12070-021-02689-4.
29. de Souza, E.C.; de Campos, V.E.; Duker, J.S. Atypical Unilateral Multifocal Choroiditis in a COVID-19 Positive Patient. *American Journal of Ophthalmology Case Reports* **2021**, *22*, 101034, doi:10.1016/j.ajoc.2021.101034.
30. Zhou, S.; Jones-lopez, E.C.; Soneji, D.J.; Azevedo, C.J.; Patel, V.R. Myelin Oligodendrocyte Glycoprotein Antibody –. **2020**, 398–402.
31. Invernizzi, A.; Pellegrini, M.; Messenio, D.; Cereda, M.; Olivieri, P.; Brambilla, A.M.; Staurenghi, G. Impending Central Retinal Vein Occlusion in a Patient with Coronavirus Disease 2019 (COVID-19). *Ocular Immunology and Inflammation* **2020**, *28*, 1290–1292, doi:10.1080/09273948.2020.1807023.
32. Deane, K.; Sarfraz, A.; Sarfraz, Z.; Valentine, D.; Idowu, A.R.; Sanchez, V. Unilateral Optic Neuritis Associated with SARS-CoV-2 Infection: A Rare Complication. *American Journal of Case Reports* **2021**, *22*, 4–7, doi:10.12659/AJCR.931665.
33. Malek, I.; Sayadi, J.; Lahiani, R.; Boumediene, M.; Salah, M. ben; Jrad, M.; Khairallah, M.; Nacef, L. Acute Bilateral Blindness in a Young Covid-19 Patient with Rhino-Orbito-Cerebral Mucormycosis. *Journal of Ophthalmic Inflammation and Infection* **2021**, *11*, 10–13, doi:10.1186/s12348-021-00272-0.

34. Micieli, J.A.; Yu, C.W. Optic Neuritis Associated with SARS-CoV-2 B.1.1.7 Variant of Concern. *Canadian Journal of Neurological Sciences* **2021**, doi:10.1017/cjn.2021.183.
35. Murchison, A.P.; Sweid, A.; Dharia, R.; Theofanis, T.N.; Tjoumakaris, I.; Jabbour, P.M.; Bilyk, J.R. Monocular Visual Loss as the Presenting Symptom of COVID-19 Infection. **2020**.
36. Reich, M.; Pauleikhoff, L.; Schröter, N.; Spang, S.; Lübke, J.; Lange, C.; Lagrèze, W.A. Sehverlust Nach Assistierter Mechanischer Beatmung Aufgrund von SARS-CoV-2 - Eine Fallserie. *Deutsches Arzteblatt International* **2020**, *117*, 561–562, doi:10.3238/arztebl.2020.0561.
37. Cleo, G.; Scott, A.M.; Islam, F.; Julien, B.; Beller, E. Usability and Acceptability of Four Systematic Review Automation Software Packages: A Mixed Method Design. *Systematic Reviews* **2019**, *8*, 1–5, doi:10.1186/S13643-019-1069-6/TABLES/3.
38. Lo, C.K.L.; Mertz, D.; Loeb, M. Newcastle-Ottawa Scale: Comparing Reviewers' to Authors' Assessments. *BMC Medical Research Methodology* **2014**, *14*, 1–5, doi:10.1186/1471-2288-14-45/TABLES/1.
