# Supplementary material for: “Vision Loss” and COVID-19 Infection: A Systematic Review and Meta-Analysis
Source: Vision (Basel). 2022 Sep 23;6(4):60. doi: 10.3390/vision6040060 (PMC9589937; doi:10.3390/vision6040060)
Supplement: Supplementary file 1 [file vision-06-00060-s001.zip › Supplementary Material S2.pdf]

Supplementary material S2: Detailed Search Strategy

PubMed

Free Text
Initial search performed on 30 April 2022
Search updated on 09 June 2022
No limits applied

Supplementary Table 1. Search term and search strategy used for paper search

#	Search Term	No. Results
1	"vision"	229.496
2	"loss"	1.095.087
3	1 AND 2	31.468
4	"blindness"	51.092
5	3 OR 4	77.454
6	" COVID-19" OR "SARS-CoV-2"	265.396
7	5 AND 6	353

Controlled Vocabulary: Medical Subject Headings (MeSH)
Initial search performed on 30 April 2019
Search updated on 9 June 2022
No limits applied

#	Search Term	No. of Results
1	("Vision Loss"[MeSH]) AND "COVID-19"[MeSH]	38
2	("Blindness"[MeSH]) AND "COVID-19"[MeSH]	38
3	("Loss of vision"[MeSH]) AND " COVID-19"[MeSH]	11
4	("Poor Vision"[MeSH]) AND " COVID-19"[MeSH]	136
5	("low vision"[MeSH]) AND " COVID-19"[MeSH]	11

All MeSH search results had already been found in the free-text search.

Embase

Free Text
Initial search performed on 30 April 2022
Search updated on 09 June 2022
[embase]/lim, no other limits applied

Mapping options enabled:

- map to preferred term in Emtree
- search also as free text in all fields
- explode using narrower Emtree terms

search as broadly as possible

#	Search Term	No. of Results
1	(("vision") AND ("loss) OR ("blindness") AND (" COVID-19" OR "SARS-CoV-2")	489

Scopus

Free Text
Initial search performed on 30 April 2022
Search updated on 9 June 2022

#	Search Term	No. of Results
1	TITLE-ABS-KEY(“vision”) AND TITLE-ABS-KEY ("loss") OR (TITLE-ABS-KEY (("blindness")) AND TITLE-ABS-KEY ("COVID-19” OR“SARS-CoV-2”)	301
