# Supplementary material for: “Vision Loss” and COVID-19 Infection: A Systematic Review and Meta-Analysis
Source: Vision (Basel). 2022 Sep 23;6(4):60. doi: 10.3390/vision6040060 (PMC9589937; doi:10.3390/vision6040060)
Supplement: Supplementary file 1 [file vision-06-00060-s001.zip › Supplementary Material S4.pdf]

## Supplementary Material S4: risk of bias evaluation

## Table 1: Newcastle–Ottawa Scale for Critical Appraisal of Cross-Sectional Studies

| | | Selection | | | | Comparability | Outcome | | |
|---|---|---|---|---|---|---|---|---|---|
| Source | Study design | Representativeness of the sample | Sample size | Non-respondents | Ascertainment of exposure | Based on design and analysis | Assessment of outcome | Statistical test | Total score |
| Carcamo Garcia et al., 2021 | Cross-sectional | +1 (b) | +1 (a) | +2 (a) | 0 (c) | +1 (a) | +1 (c) | 0 (b) | 6/10 |
| Mani et al., 2022 | Cross-sectional | +1 (b) | +1 (a) | +2 (a) | 0 (c) | +1 (a) | +1 (c) | 0 (b) | 6/10 |

Representativeness of the sample: a) Truly representative of the average in the target population. b) Somewhat representative of the average in the target population; c) Selected group of usersd) No description of the sampling strategy; Sample size: a) Justified and satisfactory. b) Not justified; Non-respondents: a) Comparability between respondents and non-respondents characteristics is established, and the response rate is satisfactory. b) The response rate is unsatisfactory, or the comparability between respondents and non-respondents is unsatisfactory. c) No description of the response rate or the characteristics of the responders and the non-responders; Ascertainment of the exposure: a) Validated measurement tool. b) Non-validated measurement tool, but the tool is available or described. c) No description of the measurement tool; Comparability: 1) The subjects in different outcome groups are comparable, based on the study design or analysis. Confounding factors are controlled. a) The study controls for the most important factor (select one). b) The study control for any additional factor; Outcome: 1) Assessment of the outcome: a) Independent blind assessment; b) Record linkage; c) Self report; d) No description. 2) Statistical test: a) The statistical test used to analyze the data is clearly described and appropriate, and the measurement of the association is presented, including confidence intervals and the probability level (p value); b) The statistical test is not appropriate, not described or incomplete.

## Table 2: Joanna Briggs Institute Critical Appraisal Checklist for Case Reports

| Source | Were the criteria for inclusion in the sample clearly defined? | Were the study subjects and setting described in detail? | Was the exposure measured in a valid and reliable way? | Were objective, standard criteria used for measurement of the condition? | Were confounding factors identified? | Were strategies to deal with confounding factors stated? | Were the outcomes measured in a valid and reliable way? | Was appropriate statistical analysis used? |
|---|---|---|---|---|---|---|---|---|
| Kaya et al. 2020 | yes | no | yes | yes | no | no | yes | Not applicable |
| Selvaraj et al. 2020 | yes | no | yes | yes | no | no | yes | Not applicable |
| Reich et al 2020. | yes | no | yes | yes | yes | yes | yes | Not applicable |
| Cyr et al. 2020 | yes | yes | yes | yes | yes | yes | yes | Not applicable |
| Zhou et al. 2020 | yes | yes | yes | yes | yes | yes | yes | Not applicable |
| Benito Pascual et al. 2020 | yes | yes | yes | yes | yes | yes | yes | Not applicable |
| Khan et al.2021 | yes | yes | yes | yes | yes | yes | yes | Not applicable |
| Invernizzi et al. 2020 | yes | yes | yes | yes | yes | yes | yes | Not applicable |
| Gascon et al.2020 | yes | yes | yes | yes | yes | yes | yes | Not applicable |
| Catharino et al 2020. | yes | no | no | yes | yes | no | no | Not applicable |
| Murchison et al.2020 | yes | yes | yes | yes | yes | yes | yes | Not applicable |
| Francois et al.2020 | yes | yes | yes | yes | yes | yes | yes | Not applicable |
| Elhassan 2021 | yes | no | yes | yes | no | no | no | Not applicable |
| Liu et al. 2021 | yes | yes | yes | yes | yes | yes | yes | Not applicable |
| De Souza et al. 2021 | yes | yes | yes | yes | no | no | yes | Not applicable |
| Katti et al. 2021 | yes | yes | yes | yes | yes | yes | yes | Not applicable |
| Rodríguez-Rodríguez et al. 2021 | yes | yes | yes | yes | yes | yes | yes | Not applicable |
| Veisi et al. 2021 | yes | yes | yes | yes | yes | yes | yes | Not applicable |
| Crane et al. 2021 | yes | yes | yes | yes | yes | yes | yes | Not applicable |
| Deane et al. 2021 | yes | yes | yes | yes | yes | yes | yes | Not applicable |
| Eswaran et al. 2021 | yes | yes | yes | yes | yes | yes | yes | Not applicable |
| Clarke et al. 2021 | yes | yes | yes | yes | yes | yes | yes | Not applicable |
| Gonzalez et al. 2021 | yes | yes | yes | yes | yes | yes | yes | Not applicable |
| Atum et al. 2021 | yes | no | yes | yes | no | no | yes | Not applicable |
| Micieli et al. 2021 | yes | yes | yes | yes | yes | yes | yes | Not applicable |
| Eslamiyeh et al. 2021 | yes | yes | yes | yes | yes | yes | yes | Not applicable |
| Malek et al. 2021 | yes | yes | yes | yes | yes | yes | yes | Not applicable |

## Table 3: Quality assessment of the included case series using the Joanna Briggs Institute (JBI) Critical Appraisal Checklist for Case Series

| Source | Were there clear criteria for inclusion in the case series? | Was the condition measured in a standard, reliable wayfor all participants included in the case series? | Were valid methods used for identification of the condition for all participants included in the case series? | Did the case series have consecutive inclusion of participants? | Did the case series have complete inclusion of participants? | Was there clear reporting of the demographics of the participants in the study? | Was there clear reporting of clinical information of the participants? | Were the outcomes or follow up results of cases clearly reported? | Was there clear reporting of the presenting site(s)/clinic(s) demographic information? | Was statistical analysis appropriate? |
|---|---|---|---|---|---|---|---|---|---|---|
| Reich et al 2020. | yes | no | yes | yes | yes | yes | yes | no | no | not applicable |
| Cyr et al. 2020 | yes | yes | yes | yes | yes | yes | yes | yes | yes | not applicable |
| Veisi et al. 2021 | yes | yes | yes | yes | yes | yes | yes | yes | yes | not applicable |
